# A case of posterior and reversible encephalopathy syndrome in a patient previously undiagnosed with lupus nephritis

**DOI:** 10.1007/s13730-025-00973-8

**Published:** 2025-02-07

**Authors:** Yoichi Kadoh, Jun Yoshino, Tomohiro Oka, Kenichi Itoga, Maki Hanada, Daisuke Niino, Atsushi Nagai, Kunihiro Ichinose, Takeshi Kanda

**Affiliations:** 1 Division of Nephrology, Department of Internal Medicine, Faculty of Medicine, Shimane University, 89-1 Enya-Cho, Izumo, Shimane 693-8501 Japan; 2 The Center for Integrated Kidney Research and Advance (IKRA), Faculty of Medicine, Shimane University, Izumo, Shimane Japan; 3 Department of Functional Pathology, Faculty of Medicine, Shimane University, Izumo, Shimane Japan; 4 Department of Neurology, Faculty of Medicine, Shimane University, Izumo , Shimane Japan; 5 Department of Rheumatology, Faculty of Medicine, Shimane University, Izumo , Shimane Japan

**Keywords:** Lupus nephritis, Posterior and reversible encephalopathy syndrome (PRES), Hypertensive emergency, Systemic lupus erythematosus (SLE)

## Abstract

Posterior reversible encephalopathy syndrome (PRES) is a rare clinico-neuroradiologic disease associated with various conditions, such as hypertension, eclampsia, chronic kidney disease, and autoimmune diseases. Here, we present the case of the unusual occurrence of PRES with hypertensive emergency and renal insufficiency in a 37-year-old woman previously undiagnosed with systemic lupus erythematosus (SLE) and lupus nephritis. The patient was emergently admitted to our hospital with sudden onset of visual impairment, headache, and high blood pressure, and she was eventually diagnosed with PRES by brain magnetic resonance imaging (MRI). Her PRES-associated clinical symptoms and MRI abnormalities were improved following anti-hypertensive treatment with calcium channel blocker. A kidney biopsy revealed diffuse proliferative glomerulonephritis with a full-house immunofluorescence pattern and fibrinoid necrosis in small blood vessels, suggesting a class IV-G (A) lupus nephritis with vasculitis. The immunosuppressive therapy with intravenous methylprednisolone pulse followed by oral prednisolone, mycophenolate mofetil, and intravenous belimumab, attenuated SLE-associated clinical manifestations including butterfly rush, edema, renal dysfunction, and proteinuria. Our case highlights the need to consider PRES as an initial clinical presentation of lupus nephritis and provide the early diagnosis and timely treatment to achieve a favorable outcome.

## Introduction

Posterior reversible encephalopathy syndrome (PRES) has become increasingly recognized as a distinctive clinico-neuroradiologic diagnosis over the past few decades due to the technological advances in brain magnetic resonance imaging (MRI) [1, 2]. The typical clinical manifestations of PRES involve intense headache, seizures, and, visual impairment, and blindness. Previous studies have demonstrated the relationships between the onset of RRES and diverse conditions, such as hypertension, eclampsia, chronic kidney disease (CKD), advanced liver diseases, autoimmune diseases, and immunosuppressive or cytotoxic medications [1, 2]. As the name suggests, PRES-associated clinical symptoms and brain-imaging lesions are reversible. The prognosis of PRES is generally favorable and the management of underlying pathogenic conditions is considered to be important [2].

Systemic lupus erythematosus (SLE) is a complex, autoimmune disease characterized by chronic inflammation and damage in multiple organs, including kidney, central nerve systems, and cardiovascular system [3, 4]. There have been multiple reported cases with PRES in SLE patients, although its prevalence is less than 1% [5]. In addition, recent studies have reported risk factors for PRES associated with SLE, such as younger age, hypertension, renal insufficiency, lupus nephritis, and SLE disease activity indices [6, 7]. However, there are limited number of cases with PRES who had not been previously diagnosed with lupus nephritis. Here we report a case of the unusual occurrence of PRES with hypertensive emergency and renal insufficiency in a patient previously undiagnosed with lupus nephritis.

## Case report

A 37-year-old woman was brought to the emergency department with sudden onset of visual impairment, headache, and high blood pressure. She had been healthy and have no medical history of interest until she was diagnosed with hypertension 3 months before admission. She developed leg edema and visited a nearby hospital 2 months earlier. Urinalysis showed proteinuria and the patient was then treated with angiotensin II receptor blocker (telmisartan 20 mg). She had received a low-estrogen oral contraceptive for dysmenorrhea for approximately 3 years until she was hospitalized.

**Fig. 1:**
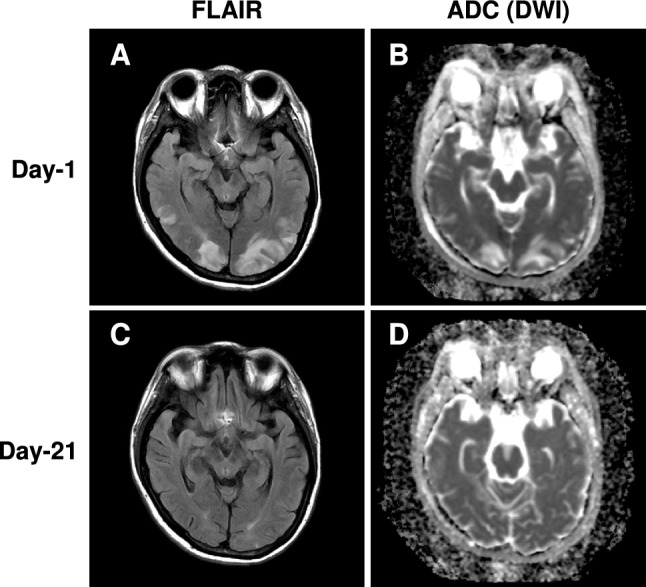
Brain magnetic resonance imaging scan. **A** The fluid attenuated inversion recovery (FLAIR) images showed high-intensity zones in the bilateral occipital lobes and cerebellum on the 1st hospital day. **B** Diffusion-weighted imaging (DWI) scans revealed increased apparent diffusion coefficient (ADC), suggesting vasogenic edema. These abnormalities were resolved on the 21st hospital day **C** and **D**

On hospitalization, the patient was awake and alert. Her height was 142 cm, body weight 49.0 kg, and body mass index 24.3 kg/m^2^. Her blood pressure was 236/122 mmHg; heart rate, 80 beats/min; respiratory rate, 16/min; body temperature, 37.2 °C; and peripheral oxygen saturation, 100%. The patient had visual impairment and intense headache. Neurologic examination revealed no other abnormalities. On physical examination, butterfly rash, and bilateral pitting edema were present. Funduscopy showed hemorrhages and exudates, suggestive of hypertensive retinopathy. The brain MRI was performed soon after admission (Fig. 1A and B). The fluid attenuated inversion recovery (FLAIR)-weighted MRI showed high-intensity zones predominantly in the bilateral occipital lobes and cerebellum (Fig. 1A). Diffusion-weighted imaging (DWI) scans revealed increased apparent diffusion coefficient (ADC), suggesting the presence of vasogenic edema (Fig. 1B). T2 star-weighted imaging indicated minute subcortical hemorrhage in the bilateral occipital lobes. Thus, the patient was diagnosed with PRES and immediately treated with calcium channel blocker (intravenous nicardipine 75–125 mg and oral amlodipine 5–10 mg followed by oral nifedipine 20–80 mg) to lower blood pressure level. Her clinical symptoms of PRES, such as headache and visual impairment, and brain-MRI lesions were improved after anti-hypertensive treatment (Fig. 1C and D). Her blood pressure was eventually controlled with oral nifedipine (80 mg) and angiotensin II receptor blocker (azilsartan 40 mg).

Laboratory tests found hemoglobin 11.5 g/dL, white blood cell count 6500/μL, platelet count 242 × 10^3^/μL, serum protein 6.5 g/dL, serum albumin 2.3 g/dL, serum lactate dehydrogenase 391 U/L, serum urea nitrogen 21 mg/dL, serum creatinine 1.35 mg/dL (estimated Glomerular Filtration Rate [eGFR] = 36.6 mL/min/1.73 m^2^), and C-reactive protein 0.78 mg/dL. Fragmented red blood cells were not detected on admission. The complement levels were low (C3 26 mg/L, C4 4 mg/L, and CH50 < 14 U/mL). Anti-nuclear (1:160), anti-double-stranded DNA (≥ 200 IU/mL), anti-SSA (≥ 1200 U/mL), anti-SSB (29.7 U/mL), and anti-cardiolipin (IgG) antibodies (32.8 U/mL) were positive, while anti-RNP, anti-Jo-1, anti-Scl −70, anti-β2 glycoprotein I (anti-β2GPI) antibodies, myeloperoxidase anti-nuclear cytoplasmic antibody (ANCA), proteinase 3-ANCA, and cryoglobulin were negative with normal activity of a disintegrin-like and metalloproteinase with thrombospondin type 1 motif 13 (ADAMTS-13). A urinalysis showed proteinuria (urinary protein/creatinine ratio [UPCR] = 3.71 g/gCr) and hematuria (20–29 red blood cells/ high-power fields. Based on the 2019 EULAR/ACR classification criteria for SLE, the patient met five criteria, accumulating a total score of 20, which confirmed the diagnosis of SLE [8].

**Fig. 2:**
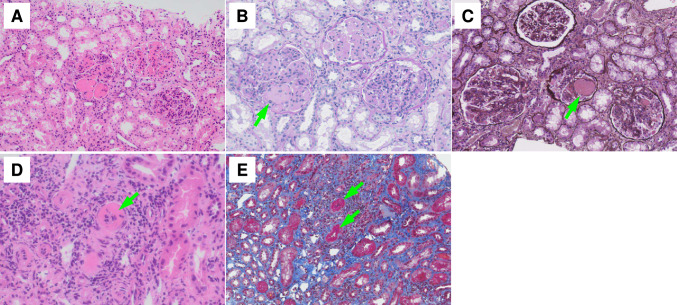
Light microscopy of the kidney biopsy. Diffuse proliferative glomerulonephritis with endocapillary proliferation, wire loop lesions, and mesangiolytic changes (arrows) (**A**, Hematoxylin–Eosin staining; **B**, Periodic acid Schiff staining; **C**, Periodic acid–methenamine silver staining). Fibrinoid necrosis in small blood vessels indicating vasculitis (arrows) (**D**, Hematoxylin–Eosin staining; **E**, Masson’s trichrome staining)

**Fig. 3:**
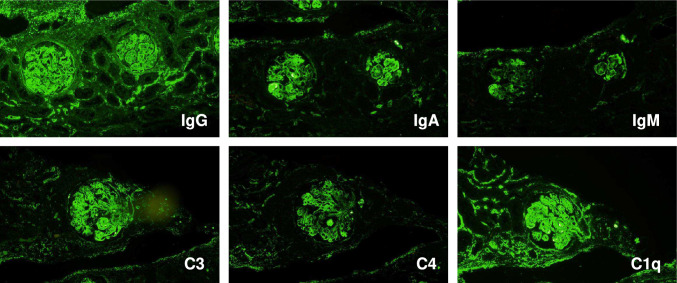
Immunofluorescent study using frozen specimen. Immunofluorescent study showed a full-house staining pattern including IgG, IgA, IgM, C3 and C1q depositions

**Fig. 4:**
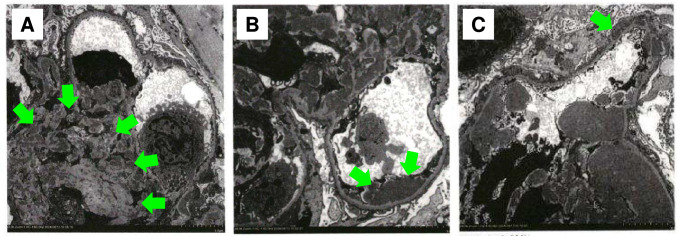
Electron microscopy. Electron-dense deposits were detected in the mesangial **A**, subendothelial **B**, and subepithelial **C** lesions (arrows)

She had worsening renal function (eGFR = 27.7 mL/min/1.73 m^2^) and proteinuria (UPCR = 7.17 g/gCr) after admission. A kidney biopsy was performed on the 10th hospital day after lowering blood pressure. It demonstrated diffuse proliferative glomerulonephritis with endocapillary proliferation, wire loop lesions, and mesangiolytic changes (Fig. 2A-C). In addition, fibrinoid necrosis was detected in small blood vessels indicating vasculitis (Fig. 2D). Thrombus formation was not evident inside the vessel lumen (Fig. 2E). An immunofluorescent study showed a full-house staining pattern including IgG, IgA, IgM, C3 and C1q depositions (Fig. 3). An electron microscopic study revealed diffuse electron-dense deposits in mesangial (Fig. 4A), subendothelial (Fig. 4B), and subepithelial (Fig. 4C) lesions. We did not detect loss of fenestrae or subendothelial edema. Taken together, these histological findings were consistent with lupus nephritis, class IV-G (A) with vasculitis.

**Fig. 5:**
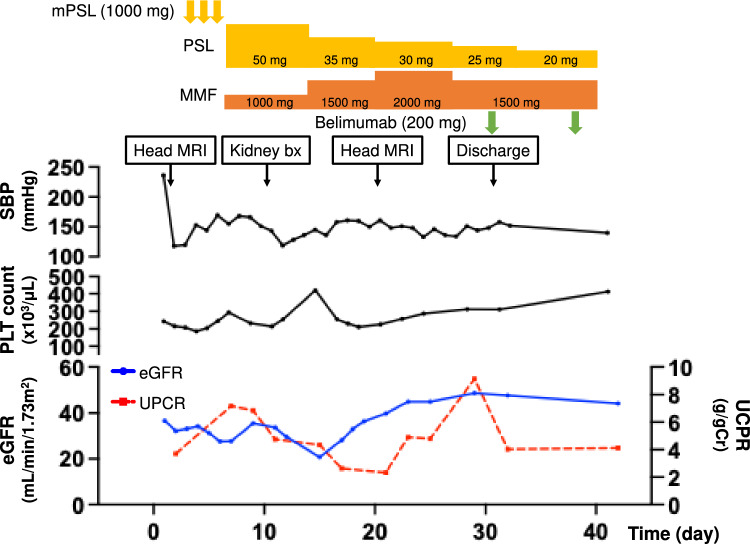
Clinical course and responses to immunosuppressive agents. mPSL, methylprednisolone; PSL, prednisolone; MMF, mycophenolate mofetil; MRI, magnetic resonance imaging; Bx, biopsy; sBP, systolic blood pressure; PLT; platelet; eGFR, estimated Glomerular Filtration Rate; UPCR, urine protein/creatinine [Cr] ratio

We initiated intravenous methylprednisolone (mPSL) pulse therapy (1000 mg/day for 3 days) from the 4th hospital day, followed by oral prednisolone (PSL) with a tapering regimen (initial dose of 50 mg/day) and mycophenolate mofetil (MMF, 1000–2000 mg/day) (Fig. 5). She was also treated with intravenous belimumab (200 mg) on the 32nd hospital day. These immunosuppressive drugs improved SLE-associated clinical manifestations including butterfly rush, edema, renal dysfunction, and proteinuria, although she did not achieve remission of proteinuria (Fig. 5). Although fragmented red blood cells (0.5–10%) were occasionally detected, thrombocytopenia was not found throughout the entire period (Fig. 5). The patient was discharged on the 33rd hospital day and she continued to receive immunosuppressive agents including PSL, MMF, and belimumab after hospital discharge.

## Discussion

We herein report a patient previously undiagnosed with lupus nephritis, who developed PRES presenting sudden onset of visual impairment, headache, and hypertensive emergency. Her PRES-associated neurological symptoms and brain-MRI abnormalities were improved following anti-hypertensive treatment with calcium channel blocker. A kidney biopsy showed a class IV-G (A) lupus nephritis with vasculitis and the immunosuppressive therapy with intravenous mPSL pulse followed by oral PSL, MMF, and intravenous belimumab, attenuated SLE-associated clinical manifestations including butterfly rush, edema, renal dysfunction, and proteinuria.

PRES has been well documented in patients with established diagnosis of SLE and lupus nephritis. However, as far as we are aware, there were only four reported cases with undiagnosed lupus nephritis who developed PRES as summarized in Table 1 [9–12]. The age at the onset of PRES was relatively young and ranged from 21 to 37 years. All cases were female and had severe hypertension. Most patients had renal insufficiency and massive proteinuria, one patient required hemodialysis, and one patient received plasma exchange. Three out of four patients underwent kidney biopsy and were diagnosed with lupus nephritis, including one class IV and two class V cases. Most cases received immunosuppressive agents, such as glucocorticoids, MMF, azathioprine, and cyclophosphamide, and hydroxycholoroquine. Taken together, age, sex, presence of hypertension, and renal insufficiency and massive proteinuria with lupus nephritis (class IV), and immunosuppressive treatment, in this case were overall similar to those in previous cases, and these clinical features are generally consistent with risk factors of PRES in patients with established diagnosis of SLE [6, 7].

**Table 1:** A summary of previous PRES cases in undiagnosed lupus nephritis

References	Age (year)	Sex	sBP/dBP (mmHg)	Serum Cr (mg/dL)	U-Pro (g/day)	SLEDAI	Biopsy (class)	RRT	PE	Immunosuppressive agent
Sulaiman et al. [9]	33	F	160/110	1.83	1.1 g	−	−	−	−	−
Hartman et al. [10]	21	F	200/108	2.4	3.5 g	−	V	−	−	mPSL pulse, PSL, MMF
De Medeiros F et al. [11]	21	F	240/110	5.74	6.2 g	22	V	HD	−	mPSL pulse, PSL, azathioprine
Wang et al. [12]	37	F	150/100	0.9	18.1 g	33	IV	−	+	mPSL pulse, cyclophosphamide pulse, hydroxycholoroquin

sBP, systolic blood pressure; dBP, diastolic blood pressure; U-Pro, urinary protein; SLEDAI, SLE disease activity index; RRT, renal replacement therapy; HD, hemodialysis; PE, plasma exchange; mPSL, methylprednisolone; PSL, prednisolone; MMF, mycophenolate mofetil

Although the overall prognosis of PRES is favorable, permanent neurological impairment and death can occur [1, 2]. Previous studies have shown that intracranial hemorrhage and brainstem involvement are major risk factors of poor clinical outcome of SLE-associated PRES, such as incomplete recovery and death [13]. More importantly, PRES is associated with a high mortality rate in SLE patients [14]. Our patient significantly improved PRES-associated clinical symptoms and brain-MRI lesions during hospitalization. Nonetheless, given the presence of subcortical hemorrhage at the diagnosis of PRES, additional follow-up brain imaging and neurological evaluation should be considered important in our case.

The underlying pathogenic mechanism of PRES remains unclear, but could be explained by rapid elevation of arterial blood pressure above the upper limit of cerebral autoregulation, leading to cerebral hyperperfusion and vasogenic edema [1, 2]. Supporting the notion, our patient developed hypertensive emergency around the time of PRES onset and anti-hypertensive therapy led to resolution of PRES-associated clinical symptoms and MRI abnormalities. SLE and lupus nephritis likely contribute to the development of hypertension through multiple complex mechanisms including chronic inflammation, oxidative stress, endothelin, renin–angiotensin system, and metabolic derangement in our case [15]. In addition, we cannot exclude the possibility that low-estrogen oral contraceptive use may have affected onset of hypertensive emergency [16]. Another possible mechanism of PRES involves endothelial damage due to systemic and local inflammatory responses associated with SLE and subsequent increased blood–brain barrier (BBB) permeability [17]. Indeed, previous studies have shown that SLE is an independent risk factor for the development of vascular inflammation and endothelial dysfunction [18, 19]. Unfortunately, we did not directly evaluate markers of systemic and local endothelial dysfunction such as circulating concentrations of tissue-type plasminogen activator, and von Willebrand factor, flow-mediated dilatation (FMD), carotid-intima media thickness, arterial stiffness and ankle-brachial index (ABI) [20]. However, given the presence of biopsy-proven renal vasculitis, our patient could have had endothelial damage in multiple organs including brain. Consistent with our findings, PRES is associated with vasculitis involving kidney including ANCA-associated vasculitis [21] and anti-glomerular basement membrane disease [22]. In addition, uremic toxins caused by renal insufficiency, a key risk factor of PRES [23], could trigger endothelial damage in our case [24]. We did not detect thrombocytopenia or pathological changes in the kidney biopsy indicating the development of thrombotic microangiopathy (TMA). Although we cannot rule out a possible sampling error associated renal biopsy, these findings indicate that TMA may not be a major contributor to systemic or renal endothelial damage in our patient. Nonetheless, elevated anti-cardiolipin antibodies may induce locally prothrombotic effects and brain endothelial dysfunction, leading to disruption of BBB [25]. Taken together, hypertension and endothelial dysfunction, two distinct but interrelated pathogenic mechanisms associated with lupus nephritis, could synergistically contribute to the development of PRES in our patient.

Recent studies found that 4–28% of patients with lupus nephritis eventually develop end-stage renal disease (ESRD) [26] and that hypertension at the onset of lupus nephritis predicts the development of CKD and ESRD [27, 28]. In addition, our patient had other risk factors of ESRD associated with lupus nephritis such as race (nonwhite), eGFR at baseline (36.6 mL/min/1.73 m^2^), hypocomplementaemia (C3 26 mg/l, C4 4 mg/l, and CH50 < 14 U/mL) and renal histology (class IV) [26, 29]. Given that our patient did not achieve remission of proteinuria following immunosuppressive therapy, long-term careful monitoring and management of disease activity in lupus nephritis are considered to be particularly necessary and important.

In conclusion, we present a case of PRES in a patient previously undiagnosed with SLE and lupus nephritis. There are various neuropsychiatric syndromes in SLE patients who may have significant overlap in neurological symptoms with PRES such as headache, seizures, epilepsy, and reduced consciousness. Thus, our case highlights the need to consider PRES as an initial clinical presentation of lupus nephritis and provide the early diagnosis and timely treatment to achieve a favorable outcome.
